# Construction of Specific Primers for Rapid Detection of South African Exportable Vegetable Macergens

**DOI:** 10.3390/ijerph121012356

**Published:** 2015-09-30

**Authors:** Bukola Rhoda Aremu, Olubukola Oluranti Babalola

**Affiliations:** 1 Department of Biological Sciences, Faculty of Agriculture, Science and Technology, North-West University, Private Bag X2046, Mmabatho 2735, South Africa; E-Mail: 23925523@nwu.ac.za; 2 Food Security and Safety Niche Area, Faculty of Agriculture, Science and Technology, North-West University, Private Bag X2046, Mmabatho 2735, South Africa

**Keywords:** ClusterW, macergens, pectolytic, rapid detection, specific primer, vegetable

## Abstract

Macergens are bacteria causing great damages to the parenchymatous tissues of vegetable both on the field and in transit. To effectively and rapidly investigate the diversity and distribution of these macergens, four specific primers were designed by retrieving 16S rDNA sequences of pectolytic bacteria from GenBank through the National Center for Biotechnology Information (NCBI). These were aligned using ClusterW via BioEdit and primers were designed using Primer3Plus platform. The size and primer location of each species and PCR product size were accurately defined. For specificity enhancement, DNA template of known macergens (*Pectobacterium chrysanthermi*) and fresh healthy vegetable were used. These primers yielded expected size of approximately 1100 bp product only when tested with known macergens and no amplicon with fresh healthy vegetable was detected. Rapid detection of macergens in rotten vegetable samples was then carried out using these primers. Nucleotide sequences of macergens identified were deposited into the GenBank and were assigned accession numbers. Hence, with these specific primers, macergens can be identified with minimal quantities of the vegetable tissues using molecular techniques, for future use of the quarantine section of the Agricultural Department of the country for quick and rapid detection of macergens before exportation.

## 1. Introduction

Several bacteria species classified to different genera that can macerate parenchymatous tissues of a wide range of plants termed macergens, can occur in growing plants and on the harvested crop either in storage or transit [1]. They cause greater losses in the production and economy of the affected plant depending on the severity of the attack [2]. The different tissue maceration enzymes produced by these macergens result in rapid tissue degradation in plants [3]. The macergens include pectolytic strains of bacteria belonging to mainly six genera namely *Erwinia*, *Xanthomonas*, *Pseudomonas*, *Clostridium*, *Cytophaga*, and *Bacillus* [4]. The activities of these macergens are tightly detrimental to agricultural efficiency and plant production, leading to greater economic losses [5].

For a complete diet, fruit and vegetables (leafy and fleshy vegetables) are highly essential, however, fruits and vegetables are being threatened by macergens both on the farm, transit and in storage, reducing their quality, yields, shelf-life and consumer satisfaction. If mistakenly eaten, it can result in food poisoning and allergens [6]. In order to guide against this, early detection of these macergens needs to be considered. Although conventional methods have been in use, they are laborious and time consuming. No diagnostic primer is yet available to discriminate macergens [7].

Polymerase Chain Reaction (PCR) assay is the most sensitive of all the existing rapid methods, to detect microbial pathogens in many specimens [8]. This involves several critical steps such as Deoxy Ribonucleic Acid (DNA) extraction, PCR amplification and the detection of amplicons through electrophoresis study. Hence, needs for rapid and accurate detection of these become imperative. Rapid detection of macergens in vegetables is becoming more critical and the development of rapid and sensitive methods is of great interest for human safety. However, molecular techniques can be used to confirm the identity and the nature of the macergens, thus the major aim of this article is to design specific primers for rapid, accurate detection and identification of macergens.

## 2. Materials and Methods

### 2.1. Primer Design

All database searching was done through the website of the National Center for Biotechnology Information (NCBI) at http://www.ncbi.nlm.nih.gov/. Sequences retrieved were as follows: *Erwinia chrysanthemi* (*E. chrysanthemi*) strain ICMP 9290 (EF530561), *E. chrysanthemi* strain Y4 (JQ867399), *E. chrysanthemi* strain 09-1 (HM222417), *E. chrysanthemi* strain H12 (GU252371), *Dickeya dadantii* (*D. dadantii*) strain SUPP2200 (AB713534), *D. dadantii* strain SUPP877 (AB713563), *D. dadantii* strain CFBP 1269 (NR_041921), *D. dadantii* strain SUPP2162 (AB713572), *D. dadantii* strain MAFF106634 (AB713545), *D. dadantii* stain MAFF301767 (AB713543), *Dickeya dieffenbachiae* (*D. dieffenbachiae*) strain LMG 25992T (JF419463), *D. dadantii* subsp. *dieffenbachiae* (JX575747), *Dickeya.* sp. 0827-3 (HQ287574), *Dickeya.* sp. strain SUPP2451 (AB713550), *Pectobacterium chrysanthemi* strain 582 (AF373175).

These pectolytic macergens 16S rDNA nucleotide sequences from NCBI were saved as FASTA files. FASTA files were copied into BioEdit files in the program BioEdit Sequence Alignments Editor, Version 7.0.9.0 [9]. Multiple Sequence alignments (MSA) were performed using the ClustalW 2.0 algorithm [10]. Stringency was varied to achieve an alignment with a small number of gaps and mismatches. Altering the stringency was also done to yield as many regions with a high degree of sequence similarity as possible. MSA’s were consolidated based on obvious discrepancies (*i.e.*, the presence of a pectolytic bacterium) and a lack of sequence similarity to the consensus. The lack of sequence similarity was measured subjectively and on a percent similarity basis when needed. Consolidated trials were then aligned with each other and sequences with low similarity were discarded.

They were then opened in BioEdit to determine the highly conserved regions where primers can be designed for macergens. The primers were designed using the Primer3Plus interface (http://frodo.wi.mit.edu/) and the best primers were selected using criteria for good primer design [11]. Before proceeding to empirical testing, the finally selected primer sequences were checked for potential hairpins structure, self-dimer, cross-dimer, and cross-homology, and tested for binding affinities to the priming sites (delta G values) using Gene infinity Platform. Their specificity was determined through *in silico* PCR in Gene Infinity platform. NCBI Blast was also used to see if the primers were able to give the target macergens. Finally, the best primers were synthesized by Integrated DNA Technology at Inqaba Biotechnical Industrial (Pty) Ltd, Pretoria, South Africa.

### 2.2. Primer Development

Primers were tested with a gradient PCR machine from 47 to 59 °C to test for varying annealing temperatures. Concentrations of MgCl_2_ were varied from 1.0 to 4.0 mM and 10 ng of DNA was used per reaction tube. Reaction volumes of 50 μL consisted of 5 μL 10 × Buffer, 10 mM dNTPs, 20 μg/mL BSA, 5 U/μL Taq polymerase, 10 μM forward and reverse primers and enough nanopure water to fill reaction volumes to 50 μL. The PCR began with a 94 °C hot start for 10 min. The PCR cycles consisted of a 94 °C melting temperature for 30 s/cycle, a 47–59 °C annealing temperature for 30 s/cycle, and a 72 °C polymerase elongation step for 1 min/cycle. The PCR ended with a 72 °C elongation for 10 min and a holding period at 4 °C for infinite time. Samples were loaded into a 1.6% agarose gel stained with EtBr (Ethidium Bromide), 1 kb DNA ladders were loaded in 5 μL volumes, while 7 μL of the sample was loaded with 2 μL of loading dye. The gel was allowed to run for 2 h at 60 V. Test results were visualized with a ChemiDoc^™^ MP System (Bio-Rad Laboratories, Hercules, CA, USA).

Primers were empirically checked for specificity, by using them to amplify a known macergens DNA template of *Pectobacterium chrysanthemi* (31 ng/μL) serving as positive control and fresh healthy vegetable DNA template as negative control. This was done in order to know if they really amplified the target region of pectolytic gene and also to eliminate any possible contamination in the PCR assay.

## 3. Detection of Macergens from Vegetable Samples

The designed primers were used for detection of macergens after the specificity test.

### 3.1. Extraction of Metagenomic DNA from Vegetables

DNA was extracted from the twenty-six rotten South African vegetables using ZR Fungal/Bacterial DNA MiniPrep^™^ (Zymo Research) according to the manufacturer instructions.

### 3.2. PCR Amplification

The average amount of the DNA used as template for PCR was 1 ng per reaction using the previously described conditions in this study. These were repeated at least twice, unless the result was not clear enough, hence were repeated three times. PCR amplicons were analyzed by electrophoresis on 1% (*w*/*v*) agarose gel as above to confirm the expected size of the amplicons and visualized using ChemiDoc Image Analyzer while remaining PCR products were purified using NucleoSpin Gel and PCR Clean-up kit (Macherey-Nagel GmbH & Co., KG Düren, Germany).

### 3.3. DNA Sequencing

The sequencing of the purified PCR products were done at Inqaba Biotechnical Industrial (Pty) Ltd, Pretoria, South Africa with PRISM^™^ Ready Reaction Dye Terminator Cycle Sequencing Kit using the dideoxy chain termination method and electrophoresed with a model ABI PRISM^®^ 3500XL DNA Sequencer (Applied Biosystems, Foster City, CA, USA) by following manufacturer’s instructions.

### 3.4. Sequence Analysis

ChromasLite version 2.33 software was used for the analysis of Chromatograms, (sense and antisense) resulting from sequencing reaction for good quality sequence assurance [12]. The resulting chromatograms were edited using BioEdit Sequence Alignment Editor [9]. After which, the resulting consensus 16S rDNA sequences obtained were Blast in the NCBI database (www.ncbi.nlm.nih.gov) with the Basic Alignment Search Tool (BLASTn) for homology in order to identify the probable organism in question [13]. These sequences were deposited in the GenBank.

### 3.5. Phylogenetic Analysis

The phylogenetic analyses based on the 16S rDNA gene for pectolytic bacteria were further used to characterize the macergens in order to establish relationship among them. The partial 16S rDNA sequences obtained for the macergens were utilized in the search of reference nucleotide sequence available in NCBI GenBank database using BlastN algorithm [13]. MAFFT version 7.0 was employed in the multiple alignment of nucleotide sequences [14], while trees were drawn based on three major techniques using MEGA 6 [15]. These techniques include: distance based (Neigbour–Joining (NJ) with cluster-based algorithm) used in calculating pairwise distance between sequences and group sequences that are most similar and character based method (Maximum Likelihood) for comparing set of data against set of models of evolution to select the best model for the variation pattern of the sequences [16].

## 4. Results and Discussions

The 16S rDNA gene of the pectolytic bacteria were best target genes for primer development because they are highly conserved regions of the bacteria and most reliable. They are present in all target organisms as single copy per genome and are improbable to undergo horizontal gene transfer. The significance of the alignment containing several pectolytic different bacterial species was that the developed primers would have a better chance of amplifying macergens community DNA as a whole. This means that our primers may encompass a broader range of species to be recognized by PCR analysis. These species could be bacteria that we had not considered during our development process. The primer sets were developed around bacterial species that can macerate plant tissues so that they could be used to amplify community DNA extracted from plant. Four primers sets were successfully developed, from the 16S sequences of the pectolytic bacteria downloaded for better performance. The designed primers tested in the Gene Infinity Platform for binding affinities to the priming sites (delta G values), showed that they did not have potential hairpin structures, self-dimer, cross-dimer and cross-homology. All the forward primer sets sequence are good due to their legitimate G/C clamp at the 3’ end, its moderate melting temperature, and its location past the 5’ end of the coding sequence. These sequences are moderate in length, which facilitate specific binding to the target gene. The *in silico* PCR performed in the Gene Infinity platform revealed an excellent specificity of designed primers. Further primer specificity, in NCBI’s Primer-BLAST also resulted in the target macergens. These are in line with the primer properties proposed by Innis and Gelfand [11] which resulted into an excellent results. Thus, generated macergens-specific PCR primers from 16S rDNA sequences of pectolytic bacteria with their properties and the locations were depicted in the Table 1.

**Table 1:** Primers properties.

Primer	Primer Set	Oligonucleotide Sequence	GC %	Tm	Length	Location	Position
M101F	Set 1	CGGACGGGTGAGTAATGTCT	55	56.5	20	16S	101-121
M1208R	Set 1	AAGGGCCATGATGACTTGAC	50	55.1	20	16S	1208-1180
M182F	Set 2	CGATCCCTAGCTGGTCTGAG	60	60.0	20	16S	182-202
M1190R	Set 2	TTATGAGGTCCGCTTGCTCT	50	60.0	20	16S	1190-1170
M180F	Set 3	GACGATCCCTAGCTGGTCTG	60	56.9	20	16S	180-200
M1190R	Set 3	TTATGAGGTCCGCTTGCTCT	50	56.0	20	16S	1190-1170
M57F	Set 4	GAGGAAGAAACCGGCGATAG	55	55.3	20	16S	57-77
M296R	Set 4	GGCGTATCCACCGATGTAAT	50	54.6	20	16S	296-279

**Figure 1:**
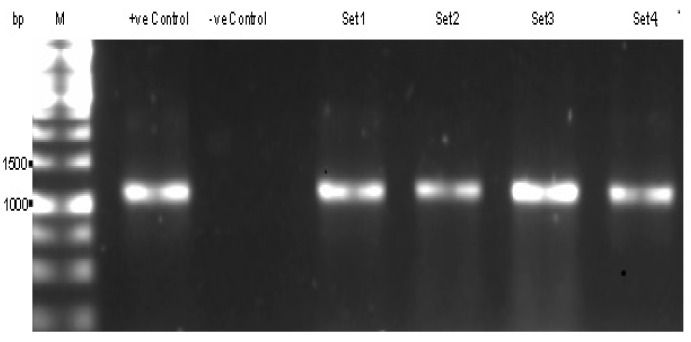
Agarose gel electrophoresis of Polymerase Chain Reaction (PCR) products of *Pectobacterium chrysanthermi* using the macergens specific primers (M101F + M1208R, M182F + M1190R, M180F + M1190R, M57F + M296R) designed in this study, which give the expected size of approximately 1100 base pairs; M: 1 kb Molecular weight marker; Lane 1: Positive Control; Lane 2: Negative Control; Lane 3: Primer Set 1 (M101F + M1208R); Lane 4: Primer Set 2 (M182F + M1190R); Lane 5: Primer Set 3 (M180F + M1190R); and Lane 6: Primer Set 4 (M57F + M296R).

In Figure 1, the sensitivities of Polymerase Chain Reaction (PCR) assay of the primers revealed that, 1000–1200 base pairs product were obtained only when macergens specific primers were used to amplify the DNA of positive control in which the vegetable were exposed to *P. chrysanthemi* and negative control that were not exposed to any macergens or microorganisms (DNA template of fresh healthy vegetable).

**Figure 2:**
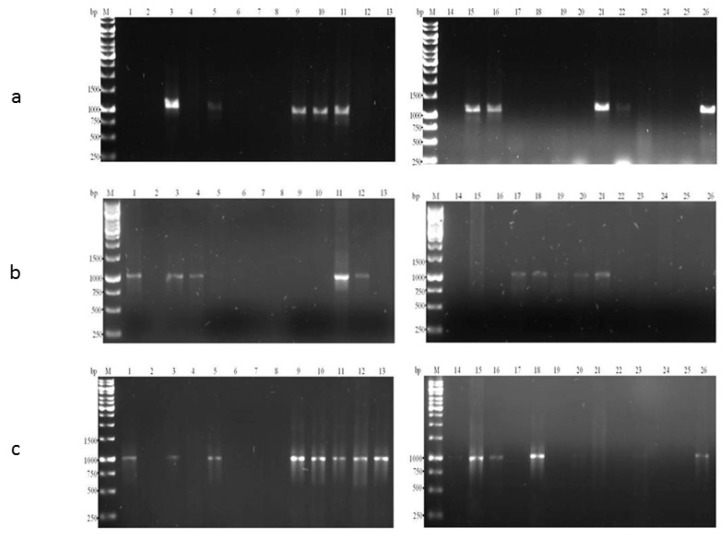
(**a**) Ethidium bromide-stained gels of Polymerase Chain Reaction (PCR) amplification products obtained from different rotten vegetable samples using set 1 and set 4 (M101F + M1208R and M57F + M296R). Lane 1, 2, 4, 6, 7, 8, 12, 13, 14, 17, 18, 19, 20, 23 and 24: No amplification; Lane 3, 5, 9, 10, 11, 15, 16, 21, 22 and 26: Amplicon size ranges from 1000 to 1100 base pairs (bp). These macergens detected are represented in Table 2. (**b**) Ethidium bromide-stained gels of PCR amplification products obtained from different rotten vegetable samples using M182F + M1190R. Lane 2, 4, 6, 7, 8, 14, 17, 19, 20, 21, 22, 23, 24 and 25: No amplification; Lane 1, 3, 5, 9, 10, 11, 12, 13, 15, 16, 18 and 26: Amplicon size of 1000 bp. These macergens detected are represented in Table 3. (**c**) Ethidium bromide-stained gels of PCR amplification products obtained from different rotten vegetable samples using M180F + M1190R. Lane 2, 5, 6, 7, 8, 9, 10, 13, 14, 15, 16, 19, 22, 23, 24, 25 and 26: No amplification; Lane 1, 3, 4, 11, 12, 17, 18, 20 and 21: Amplicon size ranges from 1000 to 1100 bp. These macergens detected are represented in Table 4.

The result obtained from the use of these specific group primers, on vegetable DNA samples revealed their ability to amplify 16S rDNA product of the correct size exclusively from DNA of these vegetables. These are depicted in Figure 2.

Hence, there is clarity in the specificity of designed primers because they did not bind to the DNA template that is devoid of the target gene in question. As a result of this, they were able to detect macergens from the vegetable samples.

**Figure 3:**
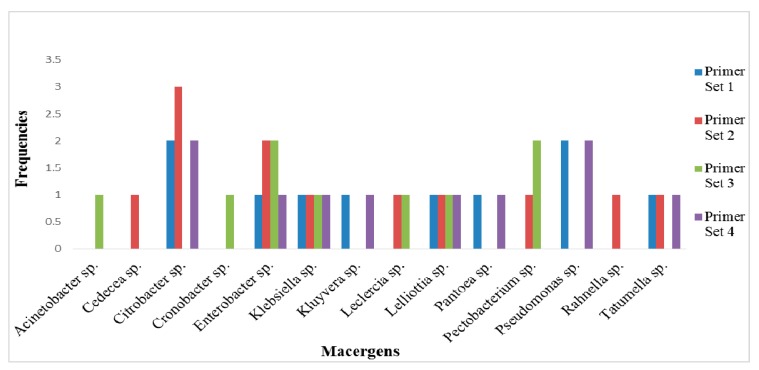
Selective frequencies of the primer with respect to the macergens in the vegetables.

With the use of these designed primers, fourteen macergens were detected in sixteen vegetables out of twenty-six samples examined. *Enterobacter* sp., *Lelliottia* sp and *Klebsiella* sp. were detected by all the primer sets. The most abundant out of all the macergens detected is *Citrobacter* sp. detected by Primer Sets 1, 2, and 4 (Figure 3).

The sequences of the macergens detected were deposited in the GenBank. In addition, the macergens detected by the primer pairs from the samples with their accession numbers are shown in Table 2, Table 3 and Table 4, respectively.

Identification of bacteria has often been difficult using traditional methods, but it has become easier with 16S rDNA sequencing [17]. Although this has insufficient discriminating power in some genera, phylogenetic analysis allows us to exclude other species and genera. This can be used to eliminate the hypothetical cause of diseases in the quarantine section. The 16S rDNA constitutes a real step forward towards accurate identification with 85.8% of species level identification, as compare to the traditional methods that are slow and unreliable [18].

**Figure 4:**
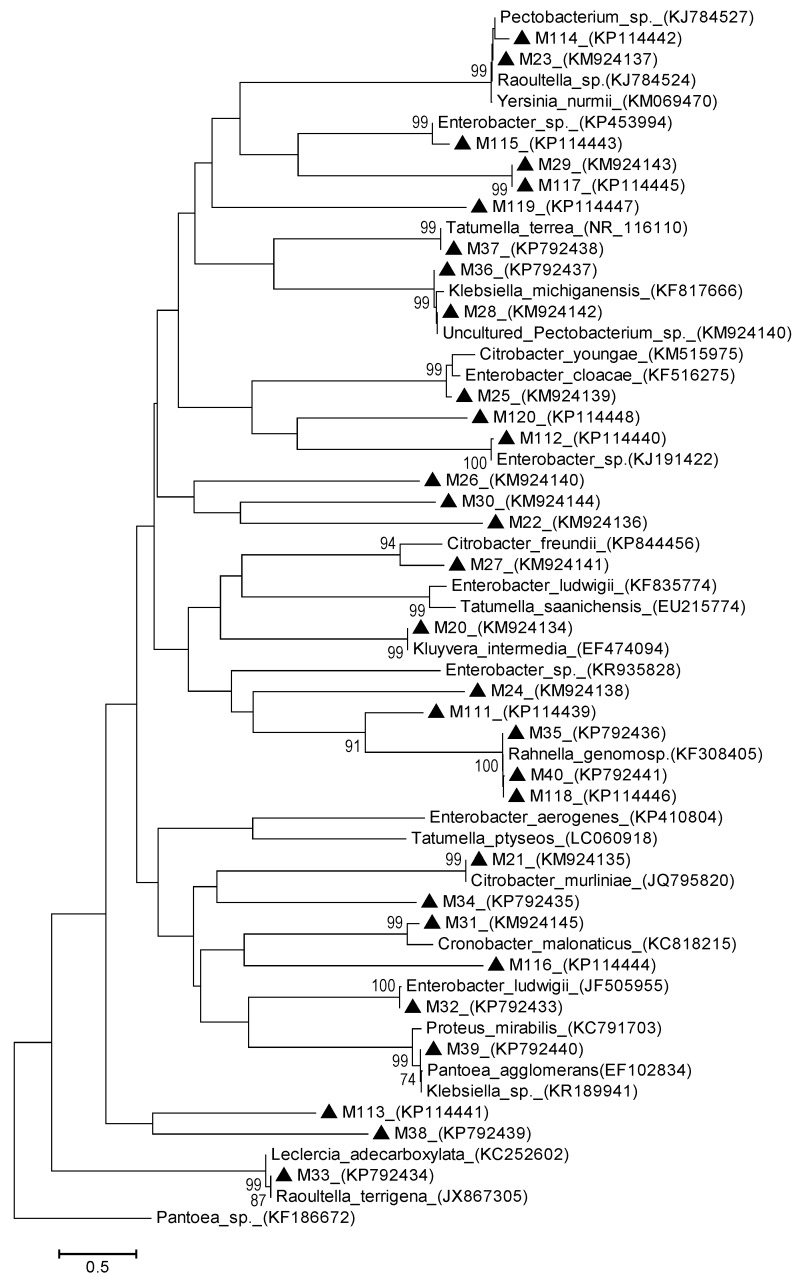
Neighbor Joining method of phylogenetic tree based on partial 16S rDNA gene sequence, showing the phylogenetic relationships between macergens and the most closely related strains from the GenBank. Numbers at the nodes indicate the levels of bootstrap support based on 1000 resampled data sets. Only values greater than 50% are shown. The scale bar indicates 0.5 base substitution per site. *Pantoea* species were set as the out-group. Sequences obtained in this study are denoted with a triangle 〔▲〕.

**Figure 5:**
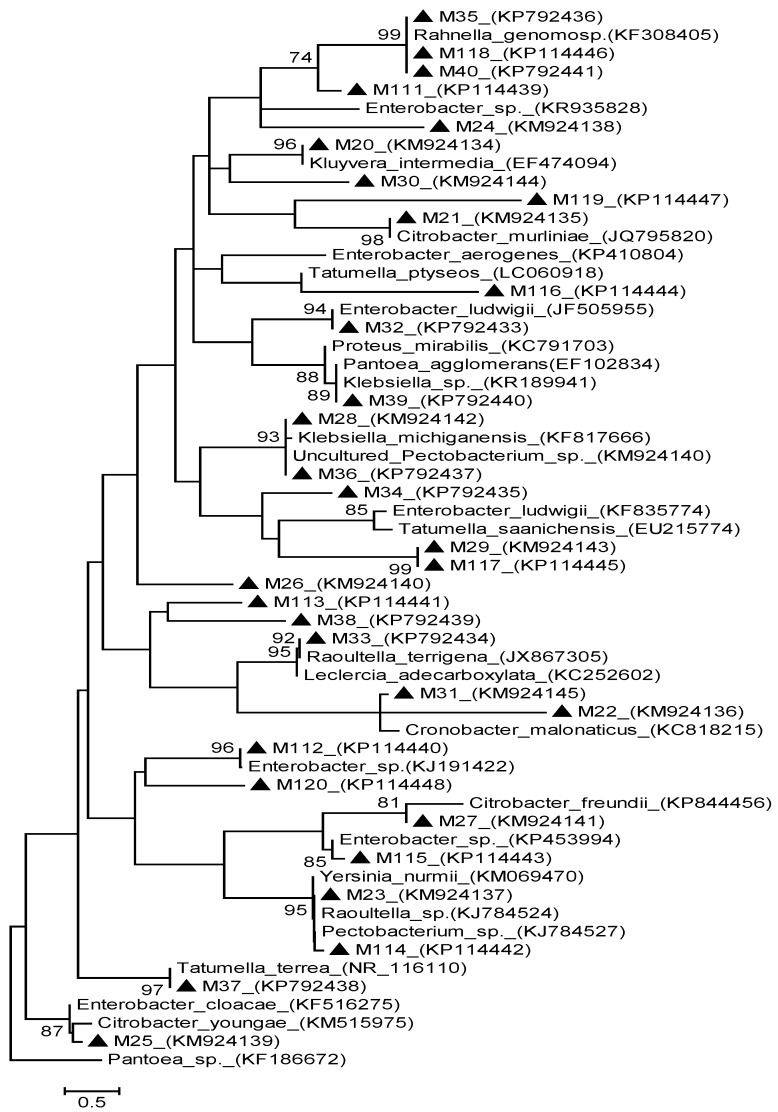
Maximum Likelihood phylogenetic tree based on partial 16S rDNA gene sequence, showing the phylogenetic relationships between macergens and the most closely related strains from the GenBank. Numbers at the nodes indicate the levels of bootstrap support based on 1000 resampled data sets. Only values greater than 50% are shown. The scale bar indicates 0.5 nucleotide substitution per site. *Pantoea* species were set as the out-group. Sequences obtained in this study are denoted with a triangle 〔▲〕.

Furthermore, Figure 4 and Figure 5 depicted the analysis on phylogenetic relationships of thirty-one sequences of macergens detected alongside with twenty-seven 16S rDNA sequences of the most closely related taxa retrieved from GenBank.

**Table 2:** Macergens detected by Set 1 and Set 4 primers from the rotten vegetables.

Lanes	Vegetable Source	Macergens	Clone Codes	Accession Number	References
3	Straight Baby Marrow	Uncultured *Kluyvera* sp.	M111	KP114439	This Study
5	White Button Mushroom	Uncultured *Enterobacter* sp.	M112	KP114440	This Study
9	Straight Small Marrow	Uncultured *Citrobacter* sp.	M113	KP114441	This Study
10	Round Baby Marrow	Uncultured *Pseudomonas* sp.	M114	KP114442	This Study
11	Red Cabbage	Uncultured *Klebsiella* sp.	M115	KP114443	This Study
15	Parsley	Uncultured *Pantoea* sp.	M116	KP114444	This Study
16	Potatoes	Uncultured *Pseudomonas* sp.	M117	KP114445	This Study
21	Spinach	Uncultured *Citrobacter* sp.	M118	KP114446	This Study
22	Spring Onions	Uncultured *Lelliottia* sp.	M119	KP114447	This Study
26	Bell Paper	Uncultured *Tatumella* sp.	M120	KP114448	This Study

**Table 3:** Macergens detected by Set 2 primers from the rotten vegetables.

Lanes	Vegetable Source	Macergens	Clone Codes	Accession Number	References
1	White Cabbage	Uncultured *Enterobacter* sp.	M20	KM924134	This Study
3	Straight Baby Marrow	Uncultured *Enterobacter* sp.	M21	KM924135	This Study
5	White Button Mushroom	Uncultured *Cedecea* sp.	M22	KM924136	This Study
9	Straight Small Marrow	Uncultured *Citrobacter* sp.	M23	KM924137	This Study
10	Round Baby Marrow	Uncultured *Citrobacter* sp.	M24	KM924138	This Study
11	Red Cabbage	Uncultured *Klebsiella* sp.	M25	KM924139	This Study
12	Iceberg Lettuce	Uncultured *Pectobacterium* sp.	M26	KM924140	This Study
13	Cauliflower	Uncultured *Citrobacter* sp.	M27	KM924141	This Study
15	Parsley	Uncultured *Leclercia* sp.	M28	KM924142	This Study
16	Potatoes	Uncultured *Rahnella* sp.	M29	KM924143	This Study
18	Potatoes	Uncultured *Lelliottia* sp.	M30	KM924144	This Study
26	Bell Pepper	Uncultured *Tatumella* sp.	M31	KM924145	This Study

**Table 4:** Macergens detected by Set 3 primers from the rotten vegetables.

Lanes	Vegetable Source	Macergens	Strains Codes	Accession Number	References
1	White Cabbage	Uncultured *Pectobacterium carotovorum*	M32	KP792433	This study
3	Straight Baby Marrow	Uncultured *Acinetobacter calcoaceticus*	M33	KP792434	This study
4	Beetroot	Uncultured *Cronobacter malonaticus*	M34	KP792435	This study
11	Red Cabbage	Uncultured *Klebsiella pneumoniae*	M35	KP792436	This study
12	Iceberg Lettuce	Uncultured *Pectobacterium* sp.	M36	KP792437	This study
17	Celery	Uncultured *Lelliottia amnigena*	M37	KP792438	This study
18	Potatoes	Uncultured *Enterobacter* sp.	M38	KP792439	This study
20	Potatoes	Uncultured *Leclercia adecarboxylata*	M39	KP792440	This study
21	Spinach	Uncultured *Enterobacter* sp.	M40	KP792441	This study

This relationship was based on two methods of phylogenetic tree namely: distance and likelihood methods. This was done in order to establish the proven resolution and statistical significance of the various treeing algorithm according to [19,20]. The distance based method inferred the evolutionary relationship using Neighbor Joining (NJ) clustered-based algorithm. The concatenated NJ showed the optimal of 46.60977 branch length with 207 position in the final dataset. Based on the cluster algorithm, NJ tree revealed the percentage of evolutionary relationship with the macergens based on the degree of differences between the sequences. The concatenated NJ showed that M32 and M112 have very high homology of 100% with *Enterobacter ludwigii* and *Enterobacter* sp., respectively. Equally, M35, M40 and M118 also shared 100% homology in NJ with *Rahnella genomosp*. In NJ, M20, M21, M31, M33, M37, M39 and M115 are closely related to *Kluyvera intermedia*, *Citrobacter murliniae*, *Cronobacter malonaticus*, *Leclercia adecarboxylata*, *Tatumella terrea* and *Enterobacter* sp., respectively, with 99% bootstrap value. Based on this distance tree, M114 and 23 have 99% homology with *Rautella* sp., *Yesina murmii* and *Pectobacterium* sp., which is a well-known primary macergens [21]. Equally, M28 and M36 also possessed 99% similarities with *Klebsiella michiganensis* and uncultured *Pectobacterium* sp. also a primary macergens [22]. In addition, M25 exhibited 99% evolutionary relationship in NJ with *Citrobacter youngae* and *Enterobacter youngae*. In NJ, M27 expressed 94% homology with *Citobacter freundii*, while M111 has 91% homology with *Rahnella genomosp*. These high bootstrap value expressed by the afore-mentioned macergens is beyond 70% borderline of degree of relatedness proposed by Wayne *et al.* [23].

In addition to this, similarities expressed by these marcergens with the reference taxa belonging to different species, is due to their high similarity value, which result in DNA reassociation values that fall below the 70% threshold values [24]. This showed high genetic relatedness that is increasingly reliable because they cannot be wiped out overnight according to Konstantinidis and Stackebrandt [19]. In NJ, M22, M24, M26, M29, M30, M38, M113, M117, M119 and M120 form distinct clades with bootstrap value less than 50% but are closer to *Enterobacter* sp. M34 and M116 also have very low bootstrap values that are less than 50% but have closest relative to be *Citrobacter murliniae* and *Cronobacter malonaticus*, respectively. These macergens did not cluster with any strains as a result of peculiarity of their nucleotide signature pattern [25]. This indicates that M22, M24, M26, M29, M30, M34, M38, M113, M116, M117, M119 and M120 are novel macergens based on their distinctness [19].

The maximum likelihood method was based on Kimura-2-parameter model [26]. This showed the relatedness of macergens based on the discrete character shared with the reference taxa. The tree with the highest log likelihood of −9764.8523 was shown with 205 final data position. This tree showed that M35, M40, and M118 are closely related to *Rahnella genomosp* with 99% bootstrap value. M111 also has a moderately similarity of 74% bootstrap value with *Rahnella genomosp*. M20 and M112 also have high homology of 96% with *Kluyvera intermedia* and *Enterobacter* sp. There is high relatedness of 98% bootstrap value between M21 and *Citrobacter murliniae*. *Enterobacter ludwigii* is closely related to M32 with 94% bootstrap value. *Pantoea agglomerans* and *Klebsiella* sp. are moderately similar to M39 with 89%, while *Proteus mirabilis* have 88% homology with M39. *Klebsiella michiganensis* and uncultured *Pectobacterium* sp. have 93% homology with M28 and M36. In addition, M33 expressed 92% similarities with *Raoultella terrigena* and *Lerclercia adecarboxylata*. Moderate relatedness of 81% was seen in M27 with *Citrobacter freundi*, 85% in M115 with *Enterobacter* sp. and 87% in M25 with *Enterobacter cloacae* and *Citrobacter youngae*. High levels of similarities of 95% were also expressed in M23 and M114 with *Yersinia murmii*, *Raoultella* sp. and *Pectobacterium* sp., respectively, and 97% in M37 with *Tatumella terrea*. All these results are still in accordance with the distance based method with the exception of M31 that clustered with 99% homology in NJ. This clustered with *Cronobacter malonaticus* in ML with a bootstrap value that is less than 50%. Hence, the relationship between *Cronobacter malonaticus* and M31 has been wiped out [19]. It is not reliable because their DNA reassociation is above the threshold level based on result depicted by ML tree [24]. ML tree also shows that some macergens did not align with any of the reference taxa based on their uniqueness, including M22, M24, M26, M29, M30, M31, M34, M38, M113, M116, M117, M119 and M120. These were classified as novel macergens with unique nucleotide signature pattern [27,28].

From the phylogenetic point of view, with the use of different algorithms, the trees inferred well-supported phylograms of macergens with high resolution of the inner branches. They all revealed that macergens are heterogeneous as they cut across different species. This is in line with [29]. Thus, it is not surprising that novel strains that do not cluster with the current known members of the previous macergens have emerged. The four oligonucleotide primer (M101F + M1208R, M182F + M1190R, M180F + M1190R, and M57F + M296R) designed in this study enhanced specificity for DNA from macergens which provides a simple method for identifying macergens.

## 5. Conclusions

The four primers designed were able to produce amplicons of expected sizes upon PCR analysis; they were optimal for heterogeneity of macergens. The high degree of similarity between the sequences chosen, through many rounds of search and refinement, implies that the primers are specific for pectolytic gene. Since these primers were designed around bacterial species, we can conclude that they must be specific to the certain amount of bacteria necessary. This method offers advantages over classical methods of detection, in the sense that the entire assay is fast, reliable, cost effective and no taxonomist is required before the identification is complete. This can be employed in analyzing and monitoring plant materials for macergens invasion in a quarantine section of the agricultural sector of a country before importation and exportation of these plants.

## Abbreviation

DNA	Deoxy Ribonucleic Acid
PCR	Polymerase Chain Reaction
RNA	Ribonucleic Acid
rDNA	*Ribosomal* Deoxy Ribonucleic Acid
rRNA	*Ribosomal* Ribonucleic Acid
